# Experimental Cross-Species Transmission of Rat Hepatitis E Virus to Rhesus and Cynomolgus Monkeys

**DOI:** 10.3390/v14020293

**Published:** 2022-01-29

**Authors:** Fengmei Yang, Yanyan Li, Yongjie Li, Weihua Jin, Suqin Duan, Hongjie Xu, Yuan Zhao, Zhanlong He, Yasushi Ami, Yuriko Suzaki, Yen Hai Doan, Naokazu Takeda, Wenjing Zhang, Masamichi Muramatsu, Tian-Cheng Li

**Affiliations:** 1 Yunnan Key Laboratory of Vaccine Research and Development on Severe Infectious Diseases, Institute of Medical Biology, Chinese Academy of Medical Sciences and Peking Union Medical College, Kunming 650118, China; yangfenmei@imbcams.com.cn (F.Y.); lyy110719@imbcams.com.cn (Y.L.); lyj981008@163.com (Y.L.); jinweihua@imbcams.com.cn (W.J.); duansuqin129@163.com (S.D.); xuhongjie@imbcams.com.cn (H.X.); zy-315@imbcams.com.cn (Y.Z.); 2 Division of Experimental Animals Research, National Institute of Infectious Diseases, Tokyo 208-0011, Japan; yami@nih.go.jp (Y.A.); ysuzaki@nih.go.jp (Y.S.); 3 Center for Emergency Preparedness and Response, National Institute of Infectious Diseases, Tokyo 208-0011, Japan; yendoan@nih.go.jp; 4 Research Institute for Microbial Diseases, Osaka University, Osaka 565-0781, Japan; seishunaotake@gmail.com; 5 Department of Virology II, National Institute of Infectious Diseases, Tokyo 208-0011, Japan; zwjviolin@foxmail.com (W.Z.); muramatsu@nih.go.jp (M.M.)

**Keywords:** rat hepatitis E virus, zoonotic infection, ALT, cross-species infection, cynomolgus monkey, rhesus monkey

## Abstract

Rat hepatitis E virus (rat HEV) was first identified in wild rats and was classified as the species *Orthohepevirus* *C* in the genera *Orthohepevirus*, which is genetically different from the genotypes HEV-1 to HEV-8, which are classified as the species *Orthohepevirus* *A*. Although recent reports suggest that rat HEV transmits to humans and causes hepatitis, the infectivity of rat HEV to non-human primates such as cynomolgus and rhesus monkeys remains controversial. To investigate whether rat HEV infects non-human primates, we inoculated one cynomolgus monkey and five rhesus monkeys with a V-105 strain of rat HEV via an intravenous injection. Although no significant elevation of alanine aminotransferase (ALT) was observed, rat HEV RNA was detected in fecal specimens, and seroconversion was observed in all six monkeys. The partial nucleotide sequences of the rat HEV recovered from the rat HEV-infected monkeys were identical to those of the V-105 strain, indicating that the infection was caused by the rat HEV. The rat HEV recovered from the cynomolgus and rhesus monkeys successfully infected both nude and Sprague-Dawley rats. The entire rat HEV genome recovered from nude rats was identical to that of the V-105 strain, suggesting that the rat HEV replicates in monkeys and infectious viruses were released into the fecal specimens. These results demonstrated that cynomolgus and rhesus monkeys are susceptible to rat HEV, and they indicate the possibility of a zoonotic infection of rat HEV. Cynomolgus and rhesus monkeys might be useful as animal models for vaccine development.

## 1. Introduction

Hepatitis E virus (HEV) is a non-enveloped positive-sense single-strand RNA virus, and it has been classified into the *Hepeviridae* family, which includes two genera—*Orthohepevirus* and *Piscihepevirus* [1]. The genus *Orthohepevirus* includes four species, *Orthohepevirus A* to *D* (HEV-A to HEV-D) [1,2]. The species HEV-A includes eight genotypes (HEV-1 to HEV-8) that have been identified in humans, monkeys, pigs, wild boars, deer, camels, mongooses, and rabbits [2,3]. The species HEV-C includes at least four genotypes (HEV-C1 to HEV-C4), and these were identified in rodents, ferrets, mink, kestrel, and foxes [4,5]. HEV-B and HEV-D were detected in birds and bats, respectively [1]. Although hepatitis E in humans is caused mainly by HEV-1 to HEV-4 infection, some cases were reported to be caused by HEV-7 or rat HEV [6,7,8]. In addition, G5 and G8 HEV also have the potential for zoonotic infection, since they are known to transmit to a non-human primate, i.e., the cynomolgus monkey [9,10,11].

Rat HEV was first identified in 2010 in wild rats in Germany [12]. Since then, rat HEV has been detected in several countries in Europe and Asia, and in the USA [13]. The main host animals are rat species (*Rattus norvegicus*, *Rattus rattus*, and others), but rat HEV sequences have also been detected in the greater bandicoot (*Bandicota indica*) and the Asian musk shrew (*Suncus murinus*) [13,14,15,16].

The rat HEV genome contains open reading frame (ORF) 1, 2 and 3 encoding a non-structural polyprotein, a capsid protein, and a small phosphoprotein, respectively, and this gene structure is commonly conserved in all HEV-related viruses [12]. The rat HEV genome also has a small ORF4 overlapping with the carboxyl terminal region of ORF1; its function is unknown [12,17]. This is different from the ORF4 demonstrated in HEV-1, which is known to enhance HEV-3 replication in vitro [18,19]. Rat HEVs are classified as genotype HEV-C1; they share only 50–60% nucleotide sequence identity with the HEV strains in species HEV-A, and they are genetically highly separated from human HEVs [12,17].

Since experimental infections of rhesus monkeys and pigs with rat HEV resulted in no sign of virus replication, rat HEV was considered not to be the source of human HEV infection [20,21]. Serological analyses of human sera indicated that a few sera showed relatively high reactivity to rat HEV, suggesting that the transmission of rat HEV-related viruses to humans was rare [22,23]. However, a rat HEV genome was detected in 2018 in a liver transplant recipient with persistent HEV infection [7]. Rat HEV was later also detected in an immunocompetent patient with severe acute hepatitis E [8], and rat HEV RNA was recently detected in Hong Kong in six of 2201 (0.27%) patients with hepatitis and 1 of 659 (0.15%) immunocompromised individuals [24]. These increasing rat HEV-related hepatitis E cases provide strong evidence of rat HEV as a potential pathogen for zoonotic infection.

In our previous study we isolated a rat HEV strain, V-105 (JX120573). The entire genome of V-105 shared 76.8%–76.9% nucleotide sequence identities with rat HEV strains from Germany, but the strain was genetically close to strain LCK-3110 (MG813927), which causes HEV infection in humans [17,25]. To investigate whether rat HEV replicates in non-human primates and to assess whether monkeys are appropriate animal models for rat HEV, we inoculated cynomolgus and rhesus monkeys with the V-105 strain of rat HEV, and the results demonstrated that these monkeys were susceptible to rat HEV.

## 2. Materials and Methods

### 2.1. The Rat HEV Strain

The rat HEV strain V-105 (GenBank accession no. JX120573) was first detected in the lung tissue of a wild rat captured in Vietnam [26]. To produce the large amounts of V-105 necessary for infection experiments, we intravenously inoculated a Wistar rat with the 10% tissue homogenate [17]. A fecal specimen was collected from the rat at day 10 post-inoculation (p.i.) and diluted with 10 mM phosphate-buffered saline (PBS) to prepare a 10% (*w*/*v*) stool suspension. The suspension was shaken at 4 °C for 1 h, clarified by centrifugation at 10,000× *g* for 30 min, and passed through a 0.45-µm membrane filter (Millipore, Bedford, MA, USA). The virus RNA titer was adjusted to 10^7^ copies/mL and stored at −80 °C until use.

### 2.2. Animals

One male 15-year-old cynomolgus monkey, M4833 (Tsukuba Primate Research Center, Tsukuba, Japan), and five female 2-year-old rhesus monkeys, M097, M107, M117, M411, and M418 (IMBCAMS, Kunming, China), were used to examine the infectivity of rat HEV. All of these monkeys were negative for rat HEV RNA by nested broad-spectrum reverse transcription-polymerase chain reaction (RT-PCR) and quantitative real-time RT-PCR (RT-qPCR) analyses, and they were all negative for anti-rat HEV IgG antibodies by a rat HEV-specific enzyme-linked immunosorbent assay (ELISA). These tests were carried out at 4 weeks and 1 week before the inoculation.

Two female 10-week-old nude rats (Long-Evans nur/nur; SLC, Hamamatsu, Japan) and three female 10-week-old Sprague-Dawley (SD) rats (IMBCAMS), all of which were negative for rat HEV RNA and anti-rat HEV IgG antibodies, were used to examine the infectivity of the rat HEV recovered from the cynomolgus and rhesus monkeys.

The nude rats and cynomolgus monkey were individually housed in Biosafety Level-2 facilities at the National Institute of Infectious Diseases (NIID), Japan. The present experiments were reviewed and approved by the institutional ethics committee of the NIID and were performed according to the Guides for Animal Experiments at the National Institute of Infectious Diseases, Tokyo, Japan under code 521005 (23 August 2020). The SD rats and rhesus monkeys were individually housed in Biosafety Level-2 facilities at the Institute of Medical Biology, Chinese Academy of Medical Science (IMBCAMS), Kunming, China. These experiments were reviewed and approved by the Ethics Committee of IMBCAMS and carried out according to the guidelines for humane treatment under codes DWSP202006-005 and DWSP202006-006 (18 June 2020).

### 2.3. Detection of Anti-Rat HEV IgG and IgM Antibodies

Anti-rat HEV IgG antibodies were detected by an ELISA using rat HEV-like particles (HEV-LPs) as the antigen and horseradish peroxidase (HRP)-conjugated goat anti-rat IgG-heavy and light-chain antibodies (Zymed Laboratories, San Francisco, CA, USA). The cut-off value for rat anti-rat HEV IgG was set at optical density (OD) values 0.181 as in a previous study [26]. HRP-conjugated goat anti-monkey IgG-heavy and light-chain antibodies (Bethyl Laboratories, Montgomery, TX, USA) and HRP-conjugated goat anti-monkey IgM (KPL, Gaithersburg, MD, USA) were used for the detection of the monkey IgG and IgM antibodies [10]. All of the HRP-conjugated antibodies were diluted 1:10,000 with PBS containing 0.05% Tween 20 (PBS-T) and 1% skim milk. To calculate the cut-off value for monkey anti-rat HEV IgG and IgM, 45 serum samples were collected from cynomolgus monkeys bred and grown at the Tsukuba Primate Research Center, Japan, and all of these sera were negative for HEV-A [27]. The OD values of anti-rat HEV IgG and IgM antibodies of these sera ranged from 0.011 to 0.169 and 0.022 to 0.184, respectively. The mean OD of anti-HEV IgG antibody in the serum samples was 0.048 with a standard deviation (SD) of 0.042, and the cut-off was calculated as 0.174 on the basis of the mean OD values plus three times the SD (0.048 + 3 × 0.042). Similarly, the mean OD of anti-HEV IgM antibody in the serum samples was 0.051 with a 0.045 SD, and the cut-off for IgM antibody was calculated as 0.186 (0.051 + 3 × 0.045). The specificity of the ELISA was examined based on the reaction between the VLPs of rat HEV and rabbit anti-VLPs of Norovirus (GII. 4), human polyomavirus JC, BK, human bocavirus, porcine bocavirus and porcine cyclevirus 2 IgG antibody. No cross-reaction was found. 

### 2.4. RT-PCR and RT-qPCR for the Detection of Rat HEV RNA

We extracted the virus RNA from 200 µL of serum and 10% stool suspensions using a MagNA Pure 96 System (Roche Diagnostics, Mannheim, Germany) with a MagNA Pure 96 DNA and Viral NA Small Volume Kit (Roche Diagnostics) according to the manufacturer’s recommendations. Reverse transcription was performed with a high-capacity cDNA reverse transcription kit (ABI Applied Biosystems, Foster City, CA, USA).

A nested broad-spectrum RT-PCR analysis was performed to amplify a portion of ORF1 as described previously [28] with slight modifications. Five microliters of the cDNA were used for the first PCR in a 50 µL reaction volume containing an external forward primer, HEV-cs (5′-TCGCGCATCACMTTYTTCCARAA-3′), and an external reverse primer, HEV-cas (5′-GCCATGTTCCAGACDGTRTTCCA-3′). Each cycle consisted of denaturation at 95 °C for 30 s, primer annealing at 52 °C for 45 s and extension at 72 °C for 60 s, followed by a final extension at 72 °C for 7 min. Two microliters of the first PCR product were used for the nested PCR with an internal forward primer, HEV-csn (5′-TGTGCTCTGTTTGGCCCNTGGTTYCDG-3′), and an internal reverse primer, HEV-casn (5′-CCAGGCTCACCR.G.ARTGYTTCTTCCA-3′). Each cycle consisted of 95 °C for 30 s, 55 °C for 45 s and 72 °C for 60 s, followed by 72 °C for 7 min. The nested PCR products were separated by electrophoresis on 2% agarose gels.

The RT-qPCR was carried out with a 7500 FAST Real-Time PCR System using TaqMan Fast Virus 1-step Master Mix (Applied Biosystems, Waltham, MA, USA). The amplification was performed under a protocol of 5 min at 50 °C, 20 s of incubation at 95 °C, followed by 40 cycles of 3 s at 95 °C and 30 s at 60 °C using a primer pair consisting of the forward primer 5′- GTGGTGCTTTTATGGTGACTG-3′ (nt 4123-4143) and reverse primer 5′- CAAACTCACTAAAATCATTCTCAAACAC-3′ (nt 4196-4223), and a probe 5′-FAM- GTTCAGGAGAAGTTCGAGGCCGCCGT-TAMRA-3′ (nt 4148-4173) [29]. A 10-fold serial dilution of the capped rat HEV RNA, 10^7^ to 10^1^ copies, was used as the standard. Amplification data were collected and analyzed with Sequence Detector software ver. 1.3 (Applied Biosystems). The detection limit was 10^3^ copies/mL.

### 2.5. Viral Genome Sequencing

The complete genome sequence of rat HEV was determined by next-generation sequencing (NGS) as described previously [10].

### 2.6. Inoculation of Monkeys and Rats, and Sample Collection

Rats and monkeys were intravenously inoculated with 10% stool suspension through the tail vein and femoral vein, respectively. The serum samples were collected weekly and used for the detection of rat HEV RNA and anti-rat HEV antibodies. The monkey serum samples were also used to examine aminotransferase (ALT) values. Fecal specimens of the rats were collected weekly, and those of the monkeys were collected daily until day 42 post-inoculation (p.i.), and then collected weekly and used for the detection of the rat HEV RNA.

### 2.7. Liver Enzyme Level

The ALT values in the sera of the cynomolgus monkey were monitored weekly using a Fuji Dri-Chem Slide GPT/ALT-PIII kit (Fujifilm, Saitama, Japan), and those in the rhesus monkey sera were monitored by an ALT detection kit (Mindray, Shenzhen, China) according to the manufacturer’s recommendations. The geometric mean titers of the ALT values over the pre-inoculation period were defined as the normal ALT value, and a twofold or greater increase at the peak was considered a sign of hepatitis.

## 3. Results

### 3.1. Rat HEV Was Transmitted to the Cynomolgus Monkey

To investigate the infectivity of rat HEV in monkeys, we intravenously inoculated one cynomolgus monkey, M4833, with 10^7^ RNA copies of the V-105 strain rat HEV. The virus RNA was first detected in the feces on day 10 p.i. with 3.68 × 10^4^ copies/g; it then gradually increased and reached a peak of 8.14 × 10^5^ copies/g on day 19 p.i. The viral RNA then gradually decreased and became undetectable from day 37 p.i.

**Figure 1:**
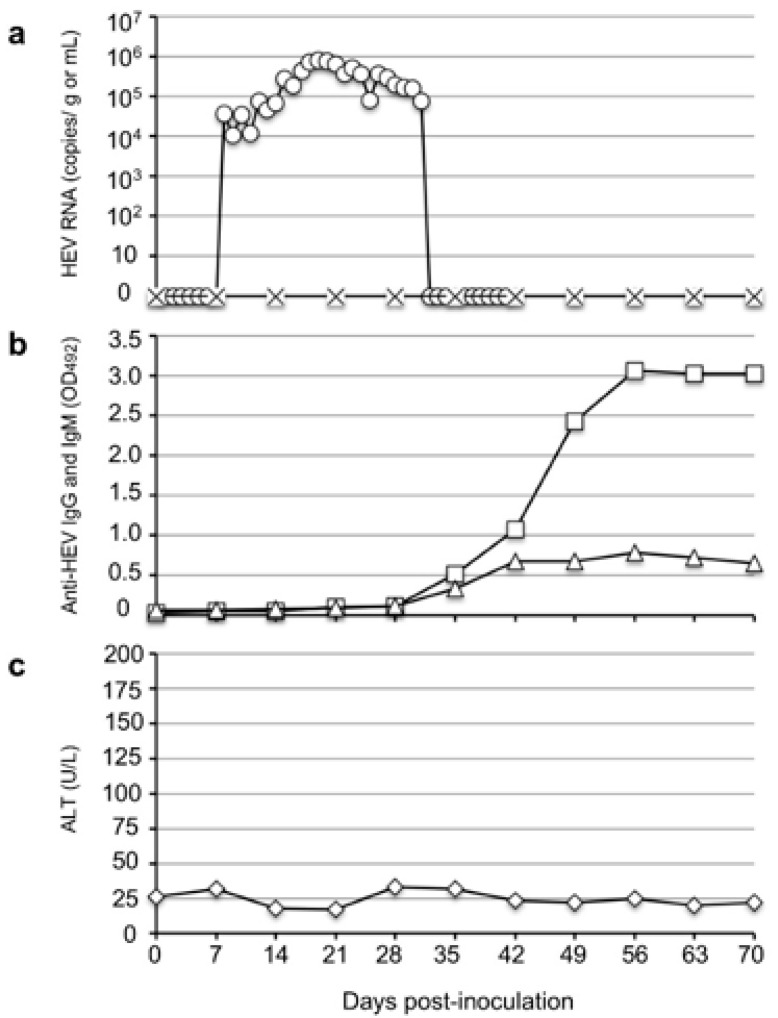
Infectivity of rat HEV in a cynomolgus monkey. A cynomolgus monkey, M4833, was intravenously inoculated with rat HEV, V-105 strain, and the kinetics of the viral RNA in the fecal (◯) and serum samples (✕) were examined by RT-qPCR (**a**). The kinetics of anti-HEV-IgG (☐) and IgM antibodies (△) in the serum samples as shown by an ELISA (**b**). The ALT values in the serum samples (◇) were measured with a Fuji Dri-Chem Slide GPT/ALT-PIII kit (**c**).

A portion of the ORF1 genome was amplified by RT-PCR using the fecal samples collected on days 15 to 19 p.i., and the nucleotide sequence analyses confirmed that 280 bp of ORF1 amplified from five fecal specimens were identical to those of the strain V-105 used for the inoculation. The viral RNA in the serum samples was under the detection limit during the observation period (Figure 1a).

The anti-rat HEV IgG and IgM antibodies were first detected on day 35 p.i. with optical density (OD) values of 0.515 and 0.333, respectively, and the same antibody levels peaked on day 56 p.i. with OD values of 3.068 and 0.791, respectively (Figure 1b). Although no ALT elevation was observed (Figure 1c), these results indicated that the rat HEV replicated in the cynomolgus monkey.

### 3.2. Rat HEV Recovered from the Cynomolgus Monkey Was Infectious

**Figure 2:**
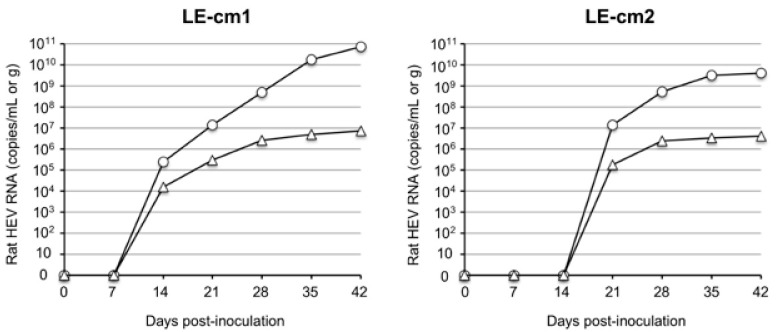
Infectivity of rat HEV recovered from the cynomolgus monkey. Two nude rats (LE-mc1 and LE-mc2) were intravenously inoculated with 0.5 mL of 10% stool suspension containing 5.2 × 10^4^ copies/mL of strain V-105 collected from monkey M4833 on day 19 p.i. The copy numbers of the virus RNA in fecal specimens (◯) and serum (△) were measured by RT-qPCR.

To examine the infectivity of the rat HEV recovered from the infected cynomolgus monkey, we prepared a 10% stool suspension from the fecal samples collected on day 19 p.i., and we inoculated 0.5 mL of the sample containing 5.2 × 10^4^ copies/mL of the virus into two nude rats, LE-cm1 and LE-cm2, through the tail vein. The viral RNAs, 2.42 × 10^5^ copies/g and 1.51 × 10^4^ copies/mL, were first detected in the feces and serum collected from nude rat LE-cm1 on day 14 p.i. Similarly, 1.38 × 10^7^ copies/g and 1.73 × 10^5^ copies/mL of the virus RNAs were detected in the feces and serum collected from nude rat LE-cm2 on day 21 p.i. The virus RNA in the feces increased to 7.34 × 10^10^ copies/g in LE-cm1 and 4.13 × 10^9^ copies/g in LE-cm2 on day 42 p.i. On the same day, 7.11 × 10^6^ copies/mL and 4.01 × 10^6^ copies/mL of the viral RNAs were detected in the serum samples collected from LE-cm1 and LE-cm2, respectively (Figure 2).

Since the monkey is not the original reservoir of rat HEV, it was important to confirm that the sequence mutation was caused during replication in the cynomolgus monkey. For this purpose, the entire viral genome sequences in the fecal samples collected from the two nude rats on day 42 p.i. were analyzed by NGS and were observed to be identical to the genome sequence of strain V-105. These results indicated that rat HEV recovered from the cynomolgus monkey was infectious, and that cynomolgus monkeys are susceptible to rat HEV.

### 3.3. Rhesus Monkeys Were Susceptible to Rat HEV

Due to the limited number of cynomolgus monkeys, we further performed infectivity experiments with rhesus monkeys. Five rhesus monkeys were separated into two groups in which three monkeys (M097, M107, and M117) were inoculated with 10^7^ copies of strain V-105 through the femoral vein, and two monkeys (M411 and M418) were similarly inoculated with 10^6^ copies of the same strain.

**Figure 3:**
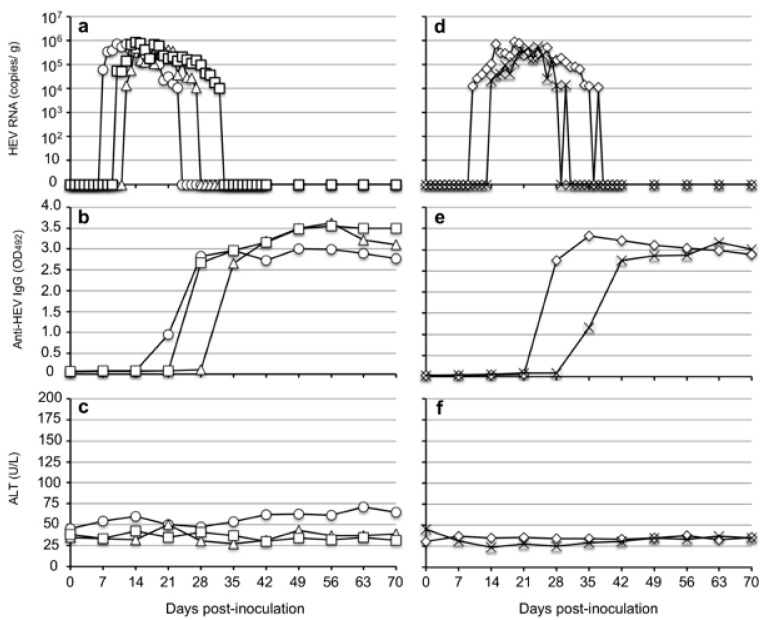
Infectivity of rat HEV in rhesus monkeys. Five rhesus monkeys were separated into two groups. Three monkeys, M097 (◯), M107 (△), and M117 (☐), were inoculated with 10^7^ copies of the rat HEV V-105 strain. Two monkeys, M411 (◇) and M418 (✕), were inoculated with 10^6^ copies of the virus. The kinetics of the viral RNA in the fecal specimens (**a**,**d**) were determined by RT-qPCR. Anti-rat HEV IgG antibodies (**b**,**e**) in the sera were determined by ELISA. ALT levels were measured by a commercial kit (**c**,**f**).

The virus RNAs were first detected in the feces of the 10^7^-copies group of three monkeys with 6.18 × 10^4^ copies/g in monkey M097 on day 7 p.i., 5.32 × 10^4^ copies/g in monkey M107 on day 10 p.i., and 1.29 × 10^4^ copies/g in M117 on day 12 p.i. The titers then gradually increased and reached peak values, but the titers were lower than 10^6^ copies/g in all three monkeys. The viral RNAs were detectable until 17 days p.i. in M097, 16 days p.i. in M107, and 23 days p.i. in M117 (Figure 3a).

Similarly, in the two monkeys inoculated with 10^6^ copies of rat HEV, the virus RNA was first detected in the feces at 1.33 × 10^4^ copies/g in monkey M411 on day 10 p.i. and at 2.08 × 10^4^ copies/g in monkey M418 on day 14. The titers then gradually increased and reached peaks, but the titers were similarly lower than 10^6^ copies/g in both monkeys. The viral RNAs were detectable until 29 days p.i. in M411 and 18 days p.i. in M418 (Figure 3d).

A portion of the ORF1 sequences was amplified by RT-PCR using the fecal samples collected from all five monkeys on day 20 p.i. The 280-nucleotide sequences derived from the monkeys were identical to those of the original sequence used for the inoculation. The viral RNA in the serum samples of all five monkeys was under the detection limit during the observation period.

Since HRP-conjugated anti-monkey IgM antibody was not available in the Kunming Laboratory, only the detection of the anti-HEV IgG antibody was carried out in the rhesus monkeys. The IgG antibodies were first detected on day 21 p.i. in M097, on day 28 p.i. in M117 and M411, and on day 35 p.i. in M107 and M418. The IgG antibodies quickly reached a plateau with OD values >3.0 (Figure 3b,e). No significant elevation of ALT was observed in all five monkeys during the period of the infection experiment (Figure 3c,f). These results indicated that the rhesus monkeys were susceptible to rat HEV and that 10^6^ copies of the rat HEV RNA was sufficient to induce infection in the monkeys.

### 3.4. The Infectivity of Rat HEV Recovered from the Rhesus Monkeys

We further examined the infectivity of the rat HEV recovered from the rhesus monkeys. Three SD rats (SD-1, SD-2, and SD-3) were intravenously inoculated through the tail vein with 0.5 mL of 10% stool suspension containing 4.8 × 10^4^ copies/mL collected from monkey M097 on day 10 p.i. The virus RNA was first detected in the rats on day 7 p.i. and peaked on day 14 p.i. at 1.0 × 10^8^ copies/g, 4.1 × 10^7^ copies/g, and 5.5 × 10^8^ copies/g in rats SD-1, SD-2 and SD-3, respectively. The virus RNA then decreased to 3.7 × 10^4^ copies/g in SD-1 and 1.90 × 10^4^ copies/g in SD-3 and became undetectable in SD-2 on day 28 p.i. Anti-HEV IgG antibodies were detected on day 14 p.i. and peaked on day 21 p.i. with OD values >3.0 in the three rats. These results indicated that the rat HEVs recovered from the rhesus monkeys were infectious and further confirmed that rhesus monkeys are susceptible to rat HEV.

## 4. Discussion

In the last decade, novel HEV strains have been discovered in various animals around the world, and it is important to determine whether these HEVs cause cross-species infection to humans. Although human hepatitis E is caused mainly by HEV-1 to -4 and HEV-7, they all belong to the species HEV-A, and evidence of zoonotic infections of rat HEV (which belongs to the species HEV-C1) is increasing. Since rat HEV is genetically diverged from HEV-A and since the pathogenicity of this virus is unclear, an animal model for infectivity and pathogenicity experiments is urgently required. Rats are a natural reservoir of rat HEV, but infected rats show no pathogenic signs [30]. It is thus necessary to investigate whether other animals (especially non-human primates) are susceptible to rat HEV and whether they can cause hepatitis.

**Figure 4:**
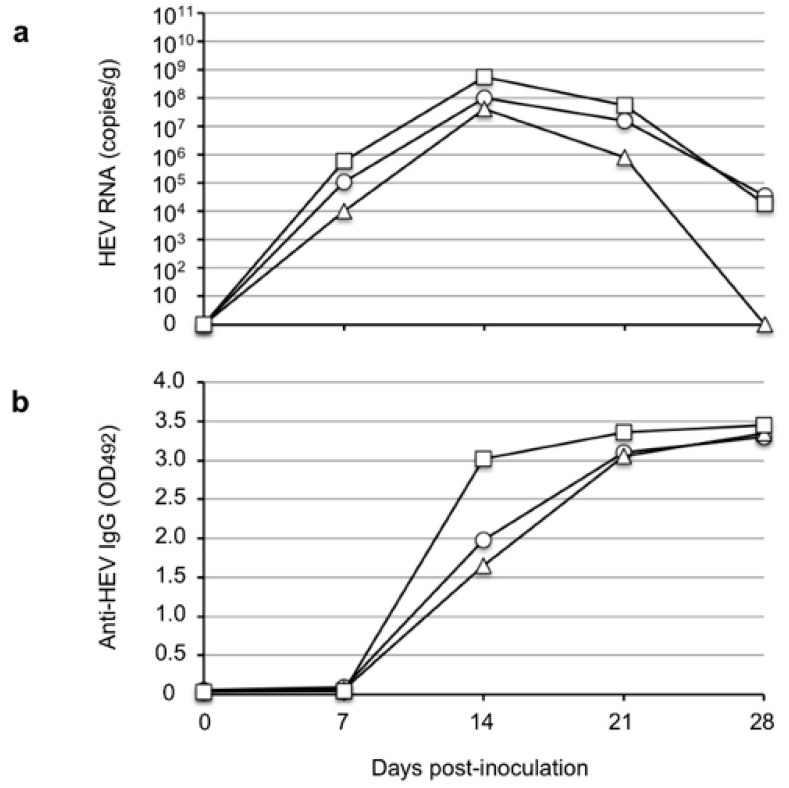
Infectivity of rat HEV recovered from the rhesus monkeys. Three SD rats, SD-1 (◯), SD-2 (△), and SD-3 (☐), were intravenously inoculated with 0.5 mL of 10% stool suspension containing 4.8 × 10^4^ copies/mL collected from monkey M097 on day 10 p.i. The virus RNAs in the fecal specimens were detected by RT-qPCR (**a**), and anti-rat HEV IgG antibodies were detected by an ELISA (**b**).

The results of the present study demonstrated that V-105 strain rat HEV was capable of experimentally infecting and replicating in cynomolgus and rhesus monkeys. These results indicate the possibility of rat HEV zoonotic infection, and they suggest that cynomolgus and rhesus monkeys might be usable as animal models for infection experiments. We observed no elevation of ALT in the infected monkeys during the period of infection, which suggests that no serious liver damage was induced in the monkeys by rat HEV infection; however, histopathological analyses will be needed to fully clarify the pathogenicity. In addition, the viral RNA in the monkey sera was under the detection limit and the titers were <10^6^ copies/g in the fecal specimens; these values are significantly lower than those in Wister rats, that is, 10^7^ copies/g to 10^8^ copies/g (Figure 4a), and those in nude rats, that is, 10^9^ copies/g to 10^10^ copies/g (Figure 2), when these species were inoculated with the same virus strain. These findings suggest that the rat HEV replication in monkeys may be limited, and they led us to examine whether the ability of the virus to replicate in humans is also limited.

Because rat HEV has the potential for zoonotic infection, the development of a vaccine and the discovery of antiviral agents are urgently needed. Our previous study demonstrated that anti-rat HEV antibodies and anti-rat HEV-LP antibodies did not neutralize HEV-3, HEV-5, or HEV-7 infection in a cell culture system, and these findings suggested that the serotype of rat HEV is different from those of other HEVs in HEV-A [9,31,32]. A recent study confirmed that an HEV vaccine, Hecolin^®^ (Xiamen Innovax Biotech, Xiamen, China), did not fully protect against rat HEV infection [25]. Taken together, the past and present results highlight the need for a vaccine against rat HEV. Our present findings demonstrated that cynomolgus and rhesus monkeys are susceptible to rat HEV infection, and they show that these monkeys can be used as a non-human primate animal model for rat HEV vaccine research. Investigations of whether rat HEV-infected rhesus monkeys are protected from human HEV infection might help clarify the serotype differences between human and rat HEV.

Since a rat HEV strain, i.e., LA-B350 (KM516906), isolated in California did not infect rhesus monkeys [20], we selected strain V-105 (which was isolated from Vietnam) to examine the infectivity of rat HEV in monkeys. Although strains LA-B350 and V-105 share 93.9% nucleotide identity, they belong to a different cluster in HEV-C1 [25]. Thirteen hepatitis E cases due to rat HEV infection have been reported to date, and most of them were in Hong Kong; genetic analyses indicated that these strains were genetically close to strain LCK-3110 (MG813927) [25]. Whether the rat HEV infection caused in Hong Kong was related to a specific variant of the virus or other local factors is not clear. Strain V-105 was genetically closest to the LCK-3110 strain group, sharing 93.7% nucleotide identity with LCK-3110. The difference in infectivity to non-human primates between strains LA-B350 and V-105 raises the question of whether monkeys are susceptible to all of the rat HEV strains in the species HEV-C1. Further studies are required to clarify the infectivity differences among rat HEV strains isolated in different areas.

## Data Availability

The sequences of HEV used in the study have been assigned (GenBank accession no. JX120573).
